# Unique Expression Pattern and Functional Role of Periostin in Human Limbal Stem Cells

**DOI:** 10.1371/journal.pone.0117139

**Published:** 2015-02-06

**Authors:** Yangluowa Qu, Wei Chi, Xia Hua, Ruzhi Deng, Jin Li, Zuguo Liu, Stephen C. Pflugfelder, De-Quan Li

**Affiliations:** 1 The Eye Institute, Xiamen University, Xiamen, China; 2 Ocular Surface Center, Cullen Eye Institute, Department of Ophthalmology, Baylor College of Medicine, Houston, Texas, United States of America; 3 Zhongshan Ophthalmic Center, State Key laboratory of Ophthalmology, Sun Yat-Sen University, Guangzhou, China; 4 Tianjin Eye Hospital, Tianjin Key Lab of Ophthalmology and Visual Science, Clinical College of Ophthalmology, Tianjin Medical University, Tianjin, China; University of Reading, UNITED KINGDOM

## Abstract

Periostin is a non-structural matricellular protein. Little is known about periostin in human limbal stem cells (LSCs). This study was to explore the unique expression pattern and functional role of periostin in maintaining the properties of human LSCs. Fresh donor corneal tissues were used to make cryosections for evaluation of periostin expression on ex vivo tissues. Primary human limbal epithelial cells (HLECs) were generated from limbal explant culture. In vitro culture models for proliferation and epithelial regeneration were performed to explore functional role of periostin in LSCs. The mRNA expression was determined by reverse transcription and quantitative real-time PCR (RT-qPCR), and the protein production and localization were detected by immunofluorescent staining and Western blot analysis. Periostin protein was found to be exclusively immunolocalized in the basal layer of human limbal epithelium. Periostin localization was well matched with nuclear factor p63, but not with corneal epithelial differentiation marker Keratin 3. Periostin transcripts was also highly expressed in limbal than corneal epithelium. In primary HLECs, periostin expression at mRNA and protein levels was significantly higher in 50% and 70% confluent cultures at exponential growth stage than in 100% confluent cultures at slow growth or quiescent condition. This expression pattern was similar to other stem/progenitor cell markers (p63, integrin β1 and TCF4). Periostin expression at transcripts, protein and immunoreactivity levels increased significantly during epithelial regeneration in wound healing process, especially in 16-24 hours at wound edge, which was accompanied by similar upregulation and activation of p63, integrin β1 and TCF4. Our findings demonstrated that periostin is exclusively produced by limbal basal epithelium and co-localized with p63, where limbal stem cells reside. Periostin promotes HLEC proliferation and regeneration with accompanied activation of stem/progenitor cell markers p63, integrin β1 and TCF4, suggesting its novel role in maintaining the phenotype and functional properties of LSC.

## Introduction

Adult stem cells, or tissue-specific stem cells, have been recognized to possess high proliferative capacity, self-renewal ability, and pluripotent potential to regenerate all cell types of the tissue where they are located [1–3]. Stem cells derived from adult tissues have the ability to regenerate tissues, and they offer great therapeutic potential for treating diseased and damaged tissues [4–7]. However, not like embryonic stem cells, consensus does not exist regarding definitive markers for these adult stem cells although a variety of molecular markers have been proposed to identify the adult stem cells.

For example, human corneal epithelial stem cells have been recognized to be located in the basal epithelial layer of the limbus, a 1.5 mm to 2 mm wide area that straddles the cornea and bulbar conjunctiva, for more than 2 decades [8–11], and thus they are referred as to limbal stem cells (LSCs). Human LSC transplantation has been successfully performed for treating ocular surface diseases with corneal epithelial stem cell deficiency, such as Stevens-Johnson syndrome, chemical, thermal and radiation injuries, extensive microbial infection, and inherited disorders such as aniridia, which are sight threatening and often cause blindness (see review [12]).

However, there are no definitive markers up to date that precisely identify LSCs although a variety of LSC markers have been proposed past years. For examples, nuclear factor p63 was previously identified as a stem cell marker for keratinocytes [13]; while later studies showed that p63 was not exclusive one and it expressed more wildly not only in LSC, but also in some transit amplifying cells, especially proliferative epithelial cells during cultures [14]. More efforts are necessary for researchers to focus on the identification of new molecular factors that maintain or determine the fate of these adult stem cells. The precise identification of the LSCs is of great importance in basic science and clinical significance. Particularly, it would largely promote the LSC-based corneal tissue engineering and improve the successful rate of LSC transplantation. Such an advance in tissue engineering is urgently needed considering an increasing shortage of corneal tissues worldwide, a critically clinical challenge to us.

Periostin, also termed osteoblast-specific factor 2, was originally found in murine osteoblasts and identified as 90 kDa protein in 1993 [15]. Periostin was then known as a matricellular protein with multiple functions in osteology, oncology, cardiovascular and respiratory systems during tissue injury, remodeling and inflammatory settings [16–18]. Interestingly, studies have shown that periostin is produced not only by stromal tissues, but also by epithelial tissues including epithelial cells in prostatic, ovarian and oral tumors [19–21]. Increasing evidence has demonstrated that periostin actively contributes to tissue injury, inflammation, fibrosis and tumor progression [22,23]. However, little is known about the functions of periostin in human LSC.

Our previous studies have identified the LSC phenotype with 3 groups of molecular markers, especially the highly expressed stem cell associated markers that maintain the properties of LSC, such as ABCG2, p63, TCF4, integrin β1, etc [24–28]. With these unique markers, we have further isolated LSC-enriched populations of corneal epithelial cells with undifferentiated phenotype, enhanced proliferative ability and tissue regenerative capacity [26,27,29,30]. Using Affymetrix whole human genome microarrays [31], we have further identified that periostin is the top 8^th^ highly expressed gene in these populations enriched in LSCs, of which the periostin mRNA levels expressed 8.63 fold higher than the differentiated corneal epithelial cells. These interesting findings encouraged us to hypothesize that periostin may have novel functional role in adult stem cells other than previously known functions. In this study, we explored the unique expression pattern and potential functions of periostin in human limbal stem cells using ex vivo donor corneal tissues and primary human limbal epithelial culture models.

## Materials And Methods

### Materials

Cell culture dishes and plates were purchased from Becton, Dickinson and Company. Dulbecco modified Eagle’s medium (DMEM), Ham F-12, gentamicin, amphotericin B, fluorescein Alexa-Fluor 488 or 594 conjugated secondary antibodies (donkey anti-goat or goat anti-mouse IgG), TaqMan Gene Expression Assays and Real-time PCR Master Mix kits were from Invitrogen Life Technologies. Fetal bovine serum (FBS) was from Hyclone. Human epidermal growth factor (EGF), hydrocortisone, cholera toxin A subunit, ITS, propidium iodide (PI) and 4',6-diamidino-2-phenylindole (DAPI) were from Sigma-Aldrich. Polyclone antibodies, rabbit anti-p63, goat anti-integrin β1 and anti-TCF4 were from Santa Cruz Biotechnology. Mouse monoclonal antibodies against keratin (K) 3, pan K, and β-actin were from Millipore, DAKO and Biolegend, respectively. The BCA protein assay kit and HRP conjugated goat anti-rabbit secondary antibody were from Thermo Fisher Scientific. Ready gels, Precision Plus Protein Unstained Standards and Precision Protein Streptactin-AP conjugate were purchase from Bio-Rad. Ready-To-Go you-Prime First-Stand Beads were purchased from GE Health Care. RNeasy Mini Kit was from Qiagen.

### Donor corneal tissues and primary human limbal epithelial cultures

Human donor corneoscleral tissues (in 72 hours postmortem), which did not meet the criteria for clinical use, from donors aged 23–64 years, were obtained from the Lions Eye Bank of Texas (Houston, TX, USA). Human tissues were handled according to the tenets of the Declaration of Helsinki. Donor corneoscleral tissues were cut through the central cornea or peripheral limbus, then frozen and sectioned using a previously described method [25,28,32]. To evaluate differential expressions, a 9-mm trephine was used to separate central cornea from the limbal tissue; and both corneal and limbal epithelia were collected by mechanical scraping, and then lysed in Qiagen RLT buffer for RNA extraction or in RIPA buffer for cellular protein extraction as described in previous publications [25, 26, 28, 29].

Primary human limbal epithelial cells (HLECs) were established from donors’ limbal explants using a previously described method [24,30]. In brief, the iris and sclera were scraped from the tissues. The limbal rings were cut into 12 pieces equally and put onto the wells of 12 well plates separately. Supplemental hormonal epithelial medium (SHEM) were used for cultures at 37°C under 5% CO_2_ and 95% humidity. The medium was changed every 2–3 day after the outgrowth emerged.

### Culture models of limbal epithelial proliferation and regeneration

To evaluate the role of periostin in proliferation, different growth stage of HLECs were carefully observed by controlling the cell confluent conditions. The cell cultures were ended when cell growth reached around 50%, 70% and 100% confluence, respectively, in 12-well plates or 8-chamber slides for different assessments.

To evaluate the role of periostin in epithelial tissue regeneration, the confluent primary HLEC cultures were wounded by creating 2mm wide area of scraped incisions across the cell layers in 6- or 12-well plates or 8-chamber slides [28,33]. The cultures were carefully observed and photographed until the wound area was completely healed in 48–72 hours. Cultures at different time periods of wound healing were used for RNA extraction, immunofluorescent staining or Western blot analysis.

### Total RNA extraction, reverse transcription (RT) and relative quantitative real-time PCR (qPCR)

Total RNA was isolated from tissues or cells, and quantified by spectrophotometry (ND-1000; NanoDrop Technologies, Wilmington, DE) using our previously described method [34,35]. The first strand cDNA was synthesized by reverse transcription from 1μg of total RNA using Ready-To-Go You-Prime First-Strand Beads. The real-time PCR was performed in the Mx3005P system (Stratagene) with a 20 μl reaction volume containing 5 μl of cDNA, 10 μl TaqMan Gene Expression Master Mix, 1 μl of TaqMan Gene Expression Assay primers and probes for periostin and glyceraldehyde-3-phosphate dehydrogenase (GAPDH). The thermocycler parameters were 50°C for 2 min, 95°C for 10 min, followed by 40 cycles of 95°C for 15 s and 60°C for 1 min. A non-template control was included to evaluate DNA contamination. The results were analyzed by the comparative threshold cycle (CT) method and normalized by a housekeeping gene GAPDH.

### Immunofluorescent staining and laser scanning confocal microscopy

Immunofluorescent staining was performed as previously described [26,29]. Corneal frozen sections or cultured corneal epithelial cells were fixed with cold methanol or freshly prepared 4% paraformaldehyde at 4°C for 10 minutes. Cultured cells were permeabilized with 0.2% Triton X-100 in phosphate-buffered saline (PBS) at room temperature for 10 minutes. After blocking with 10% normal goat or donkey serum in PBS for 30 minutes, primary antibodies against periostin (1:100), TCF4 (1:100), p63 (1:200), and integrin β1 (1:100) were applied and incubated for 2 hours at room temperature. A secondary antibody, Alexa Fluor 488-conjugated donkey anti-goat IgG (1:300) or 594-conjugated goat anti-mouse IgG (1:300), was then applied and incubated in a dark chamber for 1 hour and followed by counterstaining with a DNA-binding dye PI or DAPI (1 μg/ml in PBS) for 10 minutes. After washing with PBS, Antifade Gel/Mount and a coverslip were applied. Sections were examined and photographed with the laser scanning confocal microscopy (LSM 510, Zeiss, Thornwood, NY, http://www.zeiss.com). About 6 vision fields were photographed randomly for each culture well, and the positive and total cells were counted in the pictures. Each staining experiment was repeated at least 3–5 times.

### Western blot analysis

Western blot analysis was performed using a previous reported method [28,32]. In brief, the limbal explants were removed and the total protein of different groups of cells was extracted with RIPA buffer at the end of culture. Equal amount of protein (50 μg per lane) of every sample measured by a BCA protein assay kit, were mixed with 6×SDS reducing sample buffer and boiled for 5 minutes before loading. The proteins were separated on an SDS polyacrylamide gel and transferred electronically to PVDF membranes. The membranes were blocked with 5% nonfat milk in TTBS (50 mM Tris [pH 7.5], 0.9% NaCl, and 0.1% Tween-20) for 1 hour at room temperature and incubated with primary antibodies to periostin (1:500) and β-actin (1:1000) overnight at 4°C. After three times washes with Tris-buffered saline with 0.05% Tween 20 for 10min each, the membranes were incubated with HRP conjugated goat anti-rabbit IgG (1:1000) for 1h at room temperature. The signals were detected with a chemiluminescence reagent (ECL; GE Healthcare), and the images were acquired by a Kodak image station 2000R (Eastman Kodak, New Haven, CT).

### Statistical analysis

The Student’s t-test or analysis of variance (ANOVA) with Tukey’s post-hoc testing was used for statistical comparisons. *p* ≤ 0.05 was considered statistically significant. All of these tests were performed using the GraphPad Prism 5.0 software (Graph-Pad Prism, Inc., San Diego, CA, http://www.graphpad.com).

## Results

### Periostin was exclusively expressed by basal cells of limbal epithelium where stem cells reside

**Figure 1:**
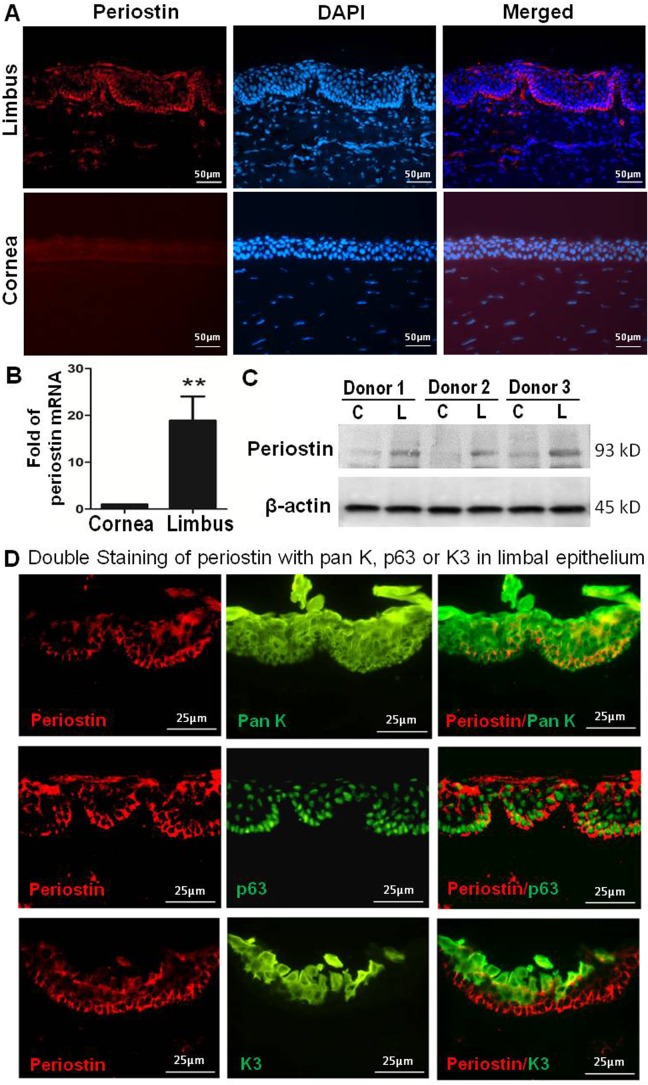
Periostin ex vivo localization in basal layer of human limbal epithelium. **(A).** Representative images showing periostin immunofluorescent staining (red) with 4',6-diamidino-2-phenylindole (DAPI, blue) counterstaining in human central and peripheral cornea and limbus. **(B).** Reverse transcription-quantitative real-time polymerase chain reaction displayed periostin mRNA expression by corneal and limbal epithelia. Data were shown as mean ± standard deviation, n = 3; **p<.01. **(C).** Western blot analysis showed significant higher levels of periostin protein in limbal epithelium (L) than that in corneal epithelium (C) from 3 donors’ tissues. **(D).** Representative images showing double immunofluorescent staining of periostin (red) with pan K, p63 or K3 (green) in limbus.

Firstly, we determined the unique expression localization of periostin in human ocular surface epithelial tissues using immune-fluorescent staining. Our results revealed that periostin was exclusively expressed by some cells in the basal player of limbal epithelial tissue, where corneal epithelial stem cells reside. As shown in Fig. 1A, periostin positive cells were primarily located in cytoplasm of basal cells, but undetectable in the suprabasal and superficial layers of limbal epithelium, nor in the all layers of central corneal epithelium. To verify this unique pattern of periostin expression in the limbal tissue, periostin expression was assayed by RT-qPCR and Western blot analysis. Our results revealed that periostin expression was much higher at the transcription levels in the limbal epithelium than in the central corneal epithelium (Fig. 1B). Western blotting further confirmed the unique expression pattern of periostin by limbal epithelium at protein levels while it was barely detectable in corneal epithelium (Fig. 1C).

### Periostin was co-localized with corneal epithelial progenitor marker p63 in the basal layer of limbal epithelium

To explore the co-localization of periostin with other epithelial markers in limbus, we performed double immunostaining assay. As shown in Fig. 1D, epithelial marker pan K stained all layers of limbal epithelium, including limbal basal layer where periostin immunoreactivity was also brightly co-localized, indicating that periostin indeed expressed by human limbal basal epithelium. Interestingly, periostin cytoplasm staining was localized in limbal basal epithelial cells where a well known keratinocyte progenitor marker p63 was expressed in the nuclear location [13,36,37]. However, periostin-positive cells appeared to be much fewer than p63-positive cells in the limbal basal layer. In contrast, the corneal epithelial specific marker K3 [24,30,38] was not detected at the basal layer of limbal epithelium where periostin positive cells located. Instead, K3 was only expressed by differentiated cells located in the superficial and partial suprabasal layers of the limbus (Fig. 1C). This co-localization pattern between periostin and p63 suggests that periostin may play a role as a potential marker for limbal stem/progenitor cells.

### Periostin expression was associated with proliferation capacity and growth stages of primary human limbal epithelial cells

**Figure 2:**
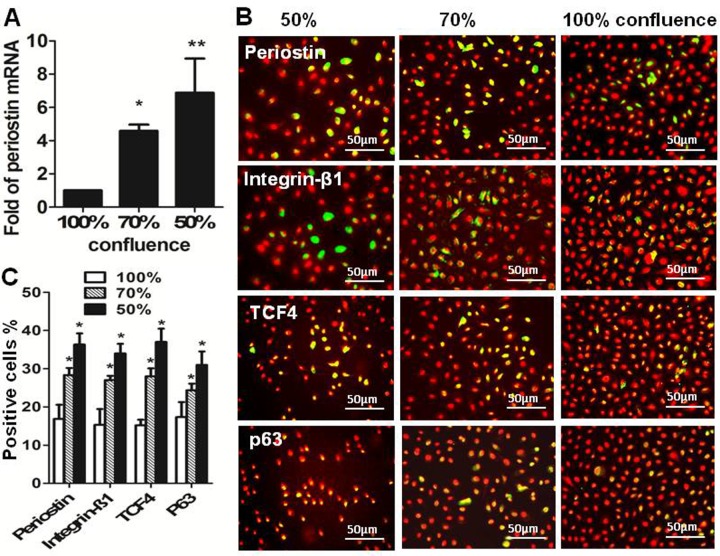
Perostin expression in human limbal epithelial cells (HLECs) at different growth stages. **(A).** Reverse transcription-quantitative real-time polymerase chain reaction displayed the expression levels (relative fold of mRNA) of periostin by 50% and 70% confluent HLECs compared with 100% confluent cells. **(B).** Representative images showing periostin, integrin β1, TCF4 and p63 immunofluorescent staining (green) with propidium iodide (PI, red) counterstaining in HLECs at different growth stages (50%, 70% and 100% confluence). **(C).** Percentages of periostin-, integrin β1-, TCF4- and p63-positive cells in 50%, 70% and 100% confluent cultures. Data shown as mean ± standard deviation, n = 3; *p<.05; **p<.01.

To evaluate the role of periostin in the proliferation and differentiation of the human limbal epithelial cells, we evaluated its potential role in primary human limbal epithelial cultures with different growth stages. Our previous studies showed that the less confluent early stage cultures at 50–70% confluence contained much more exponentially-growing cells with higher proliferative capacity than the fully confluent cultures that contained more quiescent or differentiated mature cells with low proliferative capacity [27–29]. Using in vitro proliferation culture models, our results showed that periostin mRNA expressed at significantly higher levels in cultures with early growth stages at 50% and 70% confluent conditions than that in 100% fully confluent cultures (Fig. 2A). The finding was confirmed at protein levels by immunofluorescent staining, which showed that the percentage of periostin-positive cells was 36.3±2.9 and 28.3±1.9% in the cultures at 50% and 70% confluence, respectively, significantly higher than 16.8±3.7% in fully confluent cultures (Fig. 2B and 2C).

We further evaluated the production of other progenitor cell markers, integrin β1, TCF4 and p63, which were known to play important roles in proliferation and differentiation of HLECs [13,26–28,33]. Interestingly, immunofluorescent staining revealed that the percentages of positive cells stained with integrin β1, TCF4, or p63 were also significantly higher in the cultures at 50% and 70% confluence than in 100% confluent cultures, which was similar to the expression pattern of periostin in different confluent cultures (Fig. 2B and 2C). All these results might suggest that periostin, like other corneal stem/progenitor markers, is associated with proliferation capacity of human limbal epithelial cell.

### Periostin promoted the epithelial regeneration in an in vitro wound healing model of primary human limbal epithelial cultures

**Figure 3:**
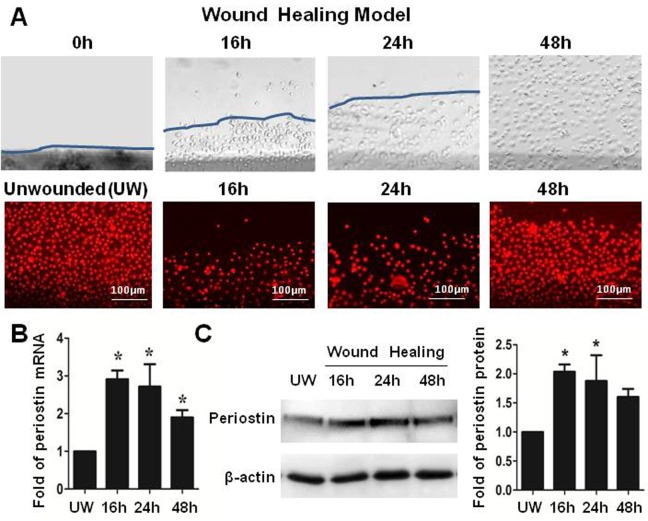
Periostin in an in vitro wound healing model of HLECs. **(A).** Representative phase images immunofluorescent staining of PI (red) showing a 2-mm wide wound area was healed within 48 hours. **(B).** Reverse transcription-quantitative real-time polymerase chain reaction data showing the expression levels (relative fold of mRNA) of periostin by HLECs at different time points after wound. **(C).** Western blot results showing the protein levels of periostin by HCECs at different time points after wound. Data were shown as mean ± standard deviation, *p<.05; **p<.01, n = 3.

**Figure 4:**
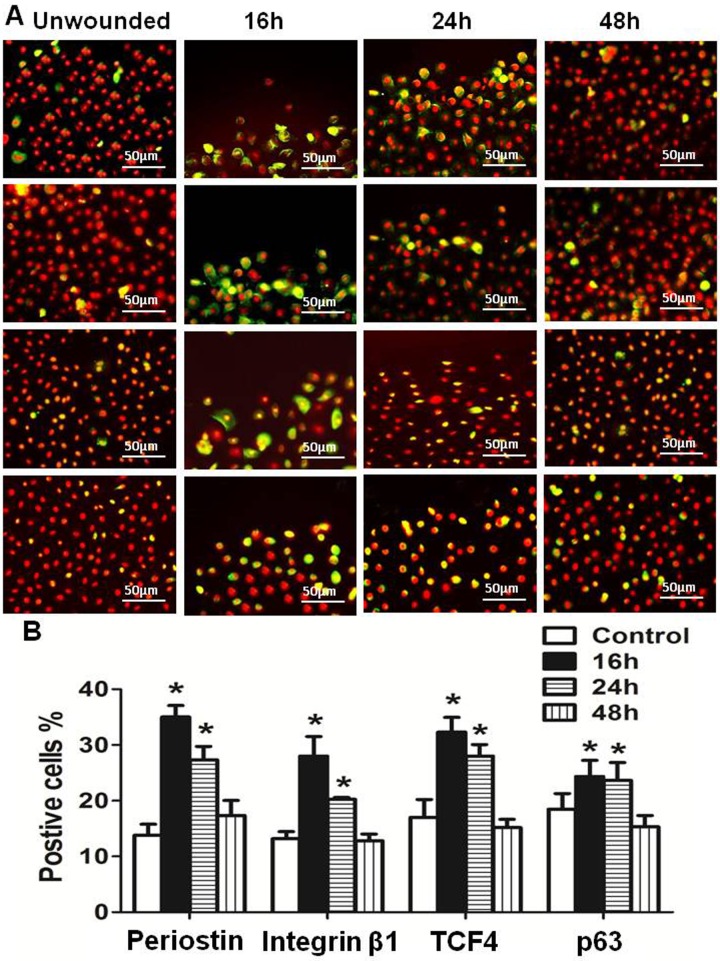
Dynamic change of periostin and other stem cell markers during limbal epithelial regeneration. **(A).** Representative images of immunofluorescent staining of periostin, integrin β1, TCF4 and p63 showing their dynamic alternation at different time points after wound. **(B).** Percentages of periostin-, integrin β1-, TCF4- and p63-positive cells at different time points in in vitro wound healing model. Data were shown as the mean ± standard deviation, *p<.05, **P<.01, n = 3.

To explore the role of periostin in regenerating corneal epithelial tissue, an in vitro wound healing model with 2mm scratched incisions was created on confluent primary limbal epithelial cultures (Fig. 3A and 3B). The dynamic expression of periostin was evaluated during the healing process until the epithelial regeneration completed. Compared with untreated culture, the expression of periostin mRNA was significantly upregulated during 48 hours of re-epithelialization process, especially in 16 to 24 hours (Fig. 3C). Western blot analysis confirmed the upregulation of periostin at protein levels during healing period (Fig. 3D). Using immunofluorescent staining, we further confirmed that the intensity of reactivity and the percentage of periostin-positive cells significantly increased, especially at the wound edge area, at 16 and 24 hours, and then reduced to near control levels (Fig. 4A and 4B). Interestingly, the similar pattern of increased immunoreactivity of integrin β1, TCF4 and p63, the well known progenitor cell markers, was observed in the wound area, especially at the wound edge during 16–24 hours of healing process. The immunofluenscent staining has lightly increase on culturs at 48h period without statistical difference from controls (Fig. 4A and 4B). These findings provided the evidence that periostin may promote the tissue regeneration via accompanied activation of other limbal stem/progenitor cells.

## Discussion

Periostin is a matricellular protein with known functions in osteology, oncology, cardiovascular and respiratory systems in tissue remodeling, tumor metastasis, and various inflammatory settings [16–18,39]. However, little is known whether periostin plays a role in maintaining the properties of ocular epithelial stem cells. The present study revealed for the first time that periostin was exclusively localized in the basal layer of human limbal epithelium where limbal stem cells reside. Periostin was detected to be well co-localized with the well-known epithelial stem/progenitor cell marker nuclear factor p63. Using different culture models of primary HLECs, we observed that periostin expression was significantly upregulated by cells in exponential growth stage rather than quiescent confluent cells, which was similar to expression patterns of other recognized stem/progenitor cell markers, integrin β1, TCF4 and p63. By creating a wound healing model in vitro, we identified that periostin upregulation promoted epithelial regeneration via accompanied activation of integrin β1, TCF4 and p63.

Different from structural extracellular matrix protein, matricellular proteins are a group of nonstructural proteins, which includes osteonectin (SPARC), CCNs, tenascins, thrombospondins, osteopontin (OPN), betaig-h3 (TGFBI) and periostin (POSTN) [16,18,40]. Periostin, encoded by gene named POSTN, also called osteoblast-specific factor 2, was originally isolated from MC3T3-E1 osteoblast cells as an important regulator of bone formation and maintenance [41,42]. As a nonstructural matricellular protein, periostin generally presents at low levels in most adult tissues; however, periostin is often highly expressed at sites of injury or inflammation and in tumors within adult organisms, indicating that the over-expression of periostin may play important role in the adhesion, invasion and angiogenesis [21,43,44].

Although periostin was reported to be detected in human cornea tissue [45], there is no report on periostin expression pattern and its function in human ocular limbal tissue where the stem cells reside. Here, we revealed that periostin was exclusively expressed by cells in the basal layer of human limbal epithelium and co-localized with nuclear factor p63. Interestingly, all periostin positive cells were co-stained with nuclear p63, but not all p63 positive cells co-stained with periostin in the basal layer of limbal epithelium. Periostin was found to be absent in the central corneal and superficial limbal epithelia, which contain more differentiated cells and express strong K3, the corneal epithelial differentiated marker.

LSCs are known to possess high proliferative potential and self-renewal capacity and are responsible for the life-long maintenance of corneal tissue [8,9,28,46]. Like other tissue-special stem cells, they can be activated by wounding or by in-vitro culture conditions to proliferate and regenerate the tissue [11,47]. In view of periostin positive cells resided in the site of stem cells, we next evaluated whether periostin endows LSCs with high proliferative and tissue regenerative capacities. Using proliferation culture model, the mRNA expression and immunoreactivity-positive cells of periostin were significantly higher by exponentially growing cells in the 50% and 70% confluent cultures than that by quiescent differentiated cells in 100% confluent cultures. Accompanied with the periostin upregulation, three known stem/progenitor cell markers, p63, TCF4, and integrin β1, were all found to be upregulated by exponentially growing cells in 50% and 70% confluent cultures. Using an in vitro wound healing, RT-qPCR and Western blotting further confirmed that both the mRNA and protein levels of periostin increased significantly in 16 to 24 hours after wound, compared with untreated controls. Strikingly, the immunofluorescent staining clearly showed the strong periostin positive cells at the edge of wound area. Interestingly, these upregulation and activation of periostin were also accompanied by the increased immunoreactivity of p63, TCF4 and integrin β1. Nuclear factor p63 has been accepted as a keratinocyte stem/progenitor cell marker associated with cell proliferation for LSCs since 2001 [13]. Integrin β1 has been used as a cell surface marker for the enrichment of putative epidermal keratinocyte stem cells [48–51] and human LSCs [27]. TCF4, a key transcription factor of Wnt signaling system, has been recently identified to be essential for maintaining LSCs via survivin, p63, p57 signaling pathway [28]. The novel findings that periostin upregulation was associated with activation of p63, integrin β1 and TCF4 in the proliferation and tissue regeneration models may suggest an important role of periostin in maintaining the phenotype and functional properties of LSCs. These novel findings were supported by a recent investigation in mouse skin, which showed a delayed re-epithelialization in periostin-deficient mice during cutaneous wound healing, indicating that periostin was essential for keratinocyte proliferation and re-epithelialization [39].

In conclusion, this study for the first time uncovered the unique expression pattern and functional role of periostin in maintaining human LSCs. Our findings demonstrated that periostin was exclusively expressed in basal layer of human limbal epithelium, and co-localized with nuclear p63. Periostin expression was upregulated in younger human limbal epithelial cells at exponential growth stage and during wound healing process, which accompanied by activation of proliferation associated markers (p63, integrin β1 and TCF4). Taken together, periostin might serve as a potential stem/progenitor cell marker that may associate with the phenotype and functional properties of limbal stem cells.

## References

[1] Diaz-Flores L Jr, Madrid JF, Gutierrez R, Varela H, Valladares F, et al (2006) Adult stem and transit-amplifying cell location. Histol Histopathol 21: 995–1027. 16763950 10.14670/HH-21.995

[2] Blau HM, Brazelton TR, Weimann JM (2001) The evolving concept of a stem cell: entity or function? Cell 105: 829–841. 11439179 10.1016/s0092-8674(01)00409-3

[3] Cotsarelis G, Kaur P, Dhouailly D, Hengge U, Bickenbach J (1999) Epithelial stem cells in the skin: definition, markers, localization and functions. Exp Dermatol 8: 80–88. 10206725 10.1111/j.1600-0625.1999.tb00351.x

[4] Borge OJ (2004) Embryonic and adult stem cells. Acta Veterinaria Scandinavica 39–43. 15347146 15347146

[5] Raff M (2003) Adult stem cell plasticity: fact or artifact? Annu Rev Cell Dev Biol 19: 1–22. 14570561 10.1146/annurev.cellbio.19.111301.143037

[6] Germain L, Carrier P, Auger FA, Salesse C, Guerin SL (2000) Can we produce a human corneal equivalent by tissue engineering? Prog Retin Eye Res 19: 497–527. 10925241 10.1016/s1350-9462(00)00005-7

[7] Jackson KA, Majka SM, Wang H, Pocius J, Hartley CJ, et al (2001) Regeneration of ischemic cardiac muscle and vascular endothelium by adult stem cells. J Clin Invest 107: 1395–1402. 11390421 10.1172/JCI12150 PMC209322

[8] Dua HS, Azuara-Blanco A (2000) Limbal stem cells of the corneal epithelium. Surv Ophthalmol 44: 415–425. 10734241 10.1016/s0039-6257(00)00109-0

[9] Tseng SC (1989) Concept and application of limbal stem cells. Eye 3 (Pt 2): 141–157. 2695347 10.1038/eye.1989.22

[10] Lavker RM, Sun TT (2000) Epidermal stem cells: properties, markers, and location. Proc Natl Acad Sci U S A 97: 13473–13475. 11087834 10.1073/pnas.250380097 PMC34083

[11] Stepp MA, Zieske JD (2005) The corneal epithelial stem cell niche. Ocul Surf 3: 15–26. 17131002 10.1016/s1542-0124(12)70119-2

[12] Dua HS, Saini JS, Azuara-Blanco A, Gupta P (2000) Limbal stem cell deficiency: concept, aetiology, clinical presentation, diagnosis and management. Indian J Ophthalmol 48: 83–92. 11116520

[13] Pellegrini G, Dellambra E, Golisano O, Martinelli E, Fantozzi I, et al (2001) p63 identifies keratinocyte stem cells. Proc Natl Acad Sci U S A 98: 3156–3161. 11248048 10.1073/pnas.061032098 PMC30623

[14] Kim SY, Cho HJ, Kim DS, Choi HR, Kwon SB, et al (2009) Differential expression of p63 isoforms in normal skin and hyperproliferative conditions. J Cutan Pathol 36: 825–830. 10.1111/j.1600-0560.2008.01167.x 19586494

[15] Takeshita S, Kikuno R, Tezuka K, Amann E (1993) Osteoblast-specific factor 2: cloning of a putative bone adhesion protein with homology with the insect protein fasciclin I. Biochem J 294 (Pt 1): 271–278. 8363580 10.1042/bj2940271 PMC1134594

[16] Norris RA, Moreno-Rodriguez R, Hoffman S, Markwald RR (2009) The many facets of the matricelluar protein periostin during cardiac development, remodeling, and pathophysiology. J Cell Commun Signal 3: 275–286. 10.1007/s12079-009-0063-5 19798597 PMC2778583

[17] Erkan M, Kleeff J, Gorbachevski A, Reiser C, Mitkus T, et al (2007) Periostin creates a tumor-supportive microenvironment in the pancreas by sustaining fibrogenic stellate cell activity. Gastroenterology 132: 1447–1464. 17408641 10.1053/j.gastro.2007.01.031

[18] Conway SJ, Izuhara K, Kudo Y, Litvin J, Markwald R, et al (2014) The role of periostin in tissue remodeling across health and disease. Cell Mol Life Sci 71: 1279–1288. 10.1007/s00018-013-1494-y 24146092 PMC3949008

[19] Tsunoda T, Furusato B, Takashima Y, Ravulapalli S, Dobi A, et al (2009) The increased expression of periostin during early stages of prostate cancer and advanced stages of cancer stroma. Prostate 69: 1398–1403. 10.1002/pros.20988 19479898

[20] Gillan L, Matei D, Fishman DA, Gerbin CS, Karlan BY, et al (2002) Periostin secreted by epithelial ovarian carcinoma is a ligand for alpha(V)beta(3) and alpha(V)beta(5) integrins and promotes cell motility. Cancer Res 62: 5358–5364. 12235007

[21] Siriwardena BS, Kudo Y, Ogawa I, Kitagawa M, Kitajima S, et al (2006) Periostin is frequently overexpressed and enhances invasion and angiogenesis in oral cancer. Br J Cancer 95: 1396–1403. 17060937 10.1038/sj.bjc.6603431 PMC2360586

[22] Liu AY, Zheng H, Ouyang G (2014) Periostin, a multifunctional matricellular protein in inflammatory and tumor microenvironments. Matrix Biol. 10.1016/j.matbio.2014.04.007 24813586

[23] Kudo A (2011) Periostin in fibrillogenesis for tissue regeneration: periostin actions inside and outside the cell. Cell Mol Life Sci 68: 3201–3207. 10.1007/s00018-011-0784-5 21833583 PMC3173633

[24] Kim HS, Jun S X, de Paiva CS, Chen Z, Pflugfelder SC, et al (2004) Phenotypic characterization of human corneal epithelial cells expanded ex vivo from limbal explant and single cell cultures. Exp Eye Res 79: 41–49. 15183099 10.1016/j.exer.2004.02.015 PMC2906376

[25] Chen Z, de Paiva CS, Luo L, Kretzer FL, Pflugfelder SC, et al (2004) Characterization of putative stem cell phenotype in human limbal epithelia. Stem Cells 22: 355–366. 15153612 10.1634/stemcells.22-3-355 PMC2906385

[26] de Paiva CS, Chen Z, Corrales RM, Pflugfelder SC, Li D-Q (2005) ABCG2 transporter identifies a population of clonogenic human limbal epithelial cells. Stem Cells 23: 63–73. 15625123 10.1634/stemcells.2004-0093 PMC2906389

[27] Li D-Q, Chen Z, Song XJ, de Paiva CS, Kim HS, et al (2005) Partial enrichment of a population of human limbal epithelial cells with putative stem cell properties based on collagen type IV adhesiveness. Exp Eye Res 80: 581–590. 15781286 10.1016/j.exer.2004.11.011 PMC2906384

[28] Lu R, Qu Y, Ge J, Zhang L, Su Z, et al (2012) Transcription factor TCF4 maintains the properties of human corneal epithelial stem cells. Stem Cells 30: 753–761. 10.1002/stem.1032 22232078 PMC5610543

[29] Chen Z, Evans WH, Pflugfelder SC, Li D-Q (2006) Gap junction protein connexin 43 serves as a negative marker for a stem cell-containing population of human limbal epithelial cells. Stem Cells 24: 1265–1273. 16424398 10.1634/stemcells.2005-0363 PMC2906383

[30] de Paiva CS, Pflugfelder SC, Li D-Q (2006) Cell size correlates with phenotype and proliferative capacity in human corneal epithelial cells. Stem Cells 24: 368–375. 16123387 10.1634/stemcells.2005-0148 PMC2906390

[31] Bian F, Liu W, Yoon KC, Lu R, Zhou N, et al (2010) Molecular signatures and biological pathway profiles of human corneal epithelial progenitor cells. Int J Biochem Cell Biol 42: 1142–1153. 10.1016/j.biocel.2010.03.022 20363357 PMC2939451

[32] Bian F, Qi H, Ma P, Zhang L, Yoon KC, et al (2010) An immunoprotective privilege of corneal epithelial stem cells against Th17 inflammatory stress by producing glial cell-derived neurotrophic factor. Stem Cells 28: 2172–2181. 10.1002/stem.539 20936708 PMC3577923

[33] Lu R, Bian F, Zhang X, Qi H, Chuang EY, et al (2011) The beta-catenin/Tcf4/survivin signaling maintains a less differentiated phenotype and high proliferative capacity of human corneal epithelial progenitor cells. Int J Biochem Cell Biol 43: 751–759. 10.1016/j.biocel.2011.01.018 21292023 PMC3131198

[34] Luo L, Li D-Q, Doshi A, Farley W, Corrales RM, et al (2004) Experimental dry eye stimulates production of inflammatory cytokines and MMP-9 and activates MAPK signaling pathways on the ocular surface. Invest Ophthalmol Vis Sci 45: 4293–4301. 15557435 10.1167/iovs.03-1145

[35] Yoon KC, de Paiva CS, Qi H, Chen Z, Farley WJ, et al (2007) Expression of Th-1 chemokines and chemokine receptors on the ocular surface of C57BL/6 mice: effects of desiccating stress. Invest Ophthalmol Vis Sci 48: 2561–2569. 17525185 10.1167/iovs.07-0002

[36] Yang A, Schweitzer R, Sun D, Kaghad M, Walker N, et al (1999) p63 is essential for regenerative proliferation in limb, craniofacial and epithelial development. Nature 398: 714–718. 10227294 10.1038/19539

[37] Di IE, Barbaro V, Ruzza A, Ponzin D, Pellegrini G, et al (2005) Isoforms of DeltaNp63 and the migration of ocular limbal cells in human corneal regeneration. Proc Natl Acad Sci U S A 102: 9523–9528. 15983386 10.1073/pnas.0503437102 PMC1172259

[38] Donisi PM, Rama P, Fasolo A, Ponzin D (2003) Analysis of limbal stem cell deficiency by corneal impression cytology. Cornea 22: 533–538. 12883346 10.1097/00003226-200308000-00009

[39] Nishiyama T, Kii I, Kashima TG, Kikuchi Y, Ohazama A, et al (2011) Delayed re-epithelialization in periostin-deficient mice during cutaneous wound healing. PLoS One 6: e18410 10.1371/journal.pone.0018410 21490918 PMC3072397

[40] Horiuchi K, Amizuka N, Takeshita S, Takamatsu H, Katsuura M, et al (1999) Identification and characterization of a novel protein, periostin, with restricted expression to periosteum and periodontal ligament and increased expression by transforming growth factor beta. J Bone Miner Res 14: 1239–1249. 10404027 10.1359/jbmr.1999.14.7.1239

[41] Bonnet N, Conway SJ, Ferrari SL (2012) Regulation of beta catenin signaling and parathyroid hormone anabolic effects in bone by the matricellular protein periostin. Proc Natl Acad Sci U S A 109: 15048–15053. 10.1073/pnas.1203085109 22927401 PMC3443161

[42] Merle B, Garnero P (2012) The multiple facets of periostin in bone metabolism. Osteoporos Int 23: 1199–1212. 10.1007/s00198-011-1892-7 22310955

[43] Ruan K, Bao S, Ouyang G (2009) The multifaceted role of periostin in tumorigenesis. Cell Mol Life Sci 66: 2219–2230. 10.1007/s00018-009-0013-7 19308325 PMC11115806

[44] Kudo Y, Siriwardena BS, Hatano H, Ogawa I, Takata T (2007) Periostin: novel diagnostic and therapeutic target for cancer. Histol Histopathol 22: 1167–1174. 17616943 10.14670/HH-22.1167

[45] Kim BY, Olzmann JA, Choi SI, Ahn SY, Kim TI, et al (2009) Corneal dystrophy-associated R124H mutation disrupts TGFBI interaction with Periostin and causes mislocalization to the lysosome. J Biol Chem 284: 19580–19591. 10.1074/jbc.M109.013607 19478074 PMC2740584

[46] Schlotzer-Schrehardt U, Kruse FE (2005) Identification and characterization of limbal stem cells. Exp Eye Res 81: 247–264. 16051216 10.1016/j.exer.2005.02.016

[47] Pajoohesh-Ganji A, Stepp MA (2005) In search of markers for the stem cells of the corneal epithelium. Biol Cell 97: 265–276. 15762848 10.1042/BC20040114

[48] Jones PH, Watt FM (1993) Separation of human epidermal stem cells from transit amplifying cells on the basis of differences in integrin function and expression. Cell 73: 713–724. 8500165 10.1016/0092-8674(93)90251-k

[49] Jones PH, Harper S, Watt FM (1995) Stem cell patterning and fate in human epidermis. Cell 80: 83–93. 7813021 10.1016/0092-8674(95)90453-0

[50] Bickenbach JR, Chism E (1998) Selection and extended growth of murine epidermal stem cells in culture. Exp Cell Res 244: 184–195. 9770361 10.1006/excr.1998.4163

[51] van Rossum MM, Schalkwijk J, van de Kerkhof PC, van Erp PE (2002) Immunofluorescent surface labelling, flow sorting and culturing of putative epidermal stem cells derived from small skin punch biopsies. J Immunol Methods 267: 109–117. 12165432 10.1016/s0022-1759(02)00151-5

